# Value of radiomics model based on enhanced computed tomography in risk grade prediction of gastrointestinal stromal tumors

**DOI:** 10.1038/s41598-021-91508-5

**Published:** 2021-06-08

**Authors:** Hairui Chu, Peipei Pang, Jian He, Desheng Zhang, Mei Zhang, Yingying Qiu, Xiaofen Li, Pinggui Lei, Bing Fan, Rongchun Xu

**Affiliations:** 1 Department of Radiology, Huzhou Central Hospital, Affiliated Central Hospital Huzhou University, Huzhou, 313000 China; 2 Department of Radiology, Huizhou Municipal Central Hospital, Huizhou, 516000 China; 3 Department of Radiology, Jiangxi Provincial People’s Hospital Affiliated to Nanchang University, Nanchang, 330006 China; 4 GE Healthcare, Hangzhou, 310000 China; 5 Department of Radiology, The Affiliated Hospital of Guizhou Medical University, Guiyang, 550000 China

**Keywords:** Cancer, Surgical oncology, Cancer, Diseases, Gastroenterology

## Abstract

To explore the application of computed tomography (CT)-enhanced radiomics for the risk-grade prediction of gastrointestinal stromal tumors (GIST). GIST patients (n = 292) confirmed by surgery or endoscopic pathology during June 2013–2019 were reviewed and categorized into low-grade (very low to low risk) and high-grade (medium to high risk) groups. The tumor region of interest (ROI) was depicted layer by layer on each patient’s enhanced CT venous phase images using the ITK-SNAP. The texture features were extracted using the Analysis Kit (AK) and then randomly divided into the training (n = 205) and test (n = 87) groups in a ratio of 7:3. After dimension reduction by the least absolute shrinkage and the selection operator algorithm (LASSO), a prediction model was constructed using the logistic regression method. The clinical data of the two groups were statistically analyzed, and the multivariate regression prediction model was constructed by using statistically significant features. The ROC curve was applied to evaluate the prediction performance of the proposed model. A radiomics-prediction model was constructed based on 10 characteristic parameters selected from 396 quantitative feature parameters extracted from the CT images. The proposed radiomics model exhibited effective risk-grade prediction of GIST. For the training group, the area under curve (AUC), sensitivity, specificity, and accuracy rate were 0.793 (95%CI: 0.733–0.854), 83.3%, 64.3%, and 72.7%, respectively; the corresponding values for the test group were 0.791 (95%CI: 0.696–0.886), 84.2%, 69.3%, and 75.9%, respectively. There were significant differences in age (t value: − 3.133, *P* = 0.008), maximum tumor diameter (Z value: − 12.163, *P* = 0.000) and tumor morphology (χ^2^ value:10.409, *P* = 0.001) between the two groups, which were used to establish a clinical prediction model. The area under the receiver operating characteristic curve of the clinical model was 0.718 (95%CI: 0.659–0.776). The proposed CT-enhanced radiomics model exhibited better accuracy and effective performance than the clinical model, which can be used for the assessment of risk grades of GIST.

## Introduction

Being the most common mesenchymal tumors of the digestive tract, gastrointestinal stromal tumors (GIST) are potential malignancies that readily metastasize to the liver and abdominal cavity with a high postoperative recurrence rate^1–3^. With reference to the 2008 National Institutes of Health (NIH) classification system, GISTs with different biological behaviors can be categorized into 4 grades: high-risk, intermediate-risk, low-risk, and very low-risk^4^. Treatment of patients with low/very low-risk GIST mainly involves surgical resection. For the early-stage and small tumors, the treatment options include minimally invasive surgery with smaller surgical wounds, rapider postoperative recovery, and fewer complications, whereas varying degrees of imatinib adjuvant treatments have been deemed necessary for high-risk tumors after surgery^5^. Therefore, the accurate diagnosis and risk stratification of GISTs before surgery is considered crucial for the determination of appropriate treatment options and patient prognosis.

With the increase in the availability of image recognition tools and the advancement in computer technology, the uses of medical images are no longer limited for visual judgment. The digital information contained therein can be mined and quantified to facilitate clinical decision-making^6^. As a novel method, radiomics has been employed to obtain these implicit data and transform them into information that can facilitate the prediction and evaluation of diseases to guide treatments approaches^7–10^. The present study aims to discuss the application of CT-enhanced radiology model for the risk-grade prediction of GIST.

## Data and method

### Clinical data

This study was approved by the ethics committee of the Huzhou Central Hospital (Affiliated Central Hospital Huzhou University), and all patients signed informed consent. A total of 384 GIST cases registered during June 2013–2019 were reviewed. A total of 37 cases with no enhanced CT examination, 45 cases with a previous treatment history, and 10 cases with poor image quality were excluded, and, finally, 292 cases were included in this study based on the following inclusion criteria:

1. Pathologically confirmed GIST patients who underwent enhanced CT examination;
2. Patients who did not receive any type of treatment (such as surgery, biopsy, radiotherapy, chemotherapy, or hormone therapy) before their enhanced CT examination;
3. Patients with high-quality CT imaging data with no artifact, where the lesion was clearly evident; and
4. Patients whose detailed pathological report with explicit risk classification was available.

### Methods

Routine plain scan and multi-phase enhanced scan were performed with the Aquilion 16 Slice Spiral CT (Toshiba, Japan) using the following scanning parameters: tube voltage: 120 kV, tube current: 150 MAS, scanning layer thickness: 0.5 mm, reconstruction layer thickness: 5 mm, layer spacing: 2 mm; 18-G venous indwelling needle embedded in the elbow vein, and non-ionic contrast agent iodofol injected at 2.5 mL/s with the injection volume of 1.5 mL/kg.

### Radiomics analysis

The Artificial Intelligence Kit (AI-Kit, Version: 3.0.1.A) was used for the analysis. Following the radiomics methods, the software delivered a series of imaging features by analyzing the heterogenicity of the target region.

#### Lesion segmentation

**Figure 1:**
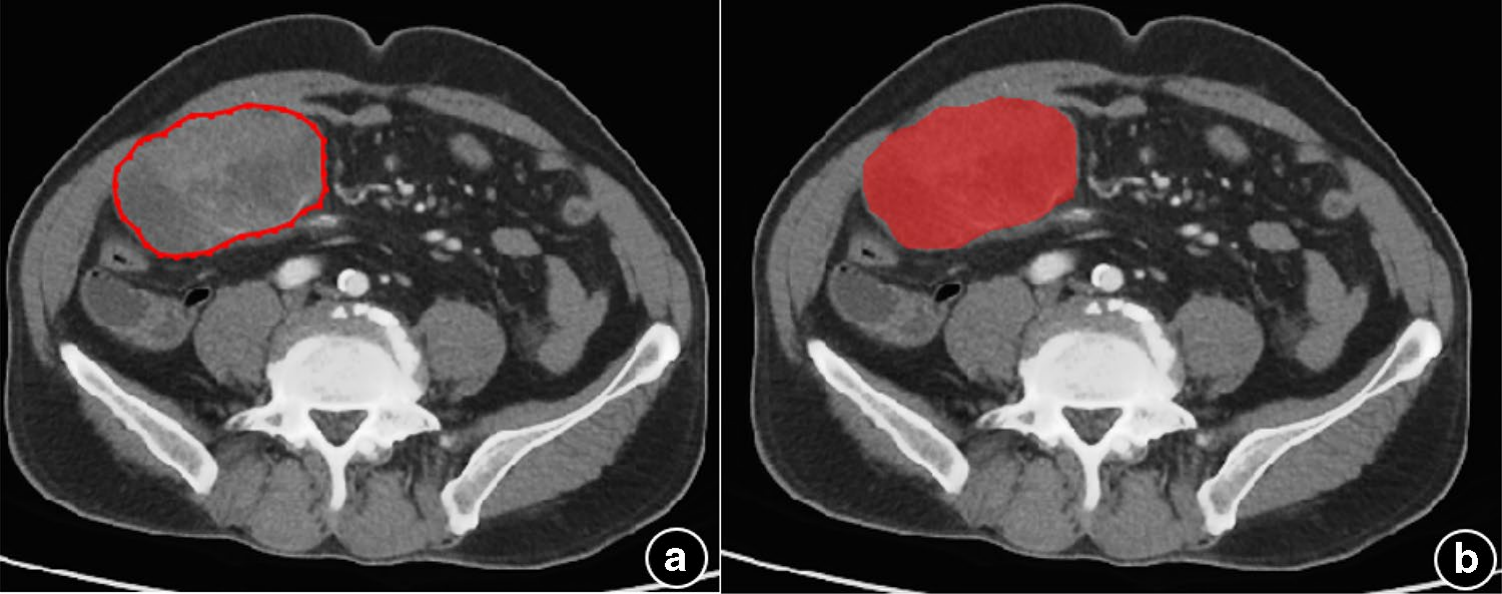
The image-processing software ITK⁃SNAP was used to manually delineate tumor ROIs along the lesion fringes on all the layers containing the tumors through the enhanced-CT venous phase images, and then the images were merged into 3D ROI images (red).

The venous phase images of all patients were imported into the image processing software ITK-SNAP (version 3.6.0, http://www.itksnap.org/)^7^ in the DICOM format. Two doctors unaware of the pathology manually delineated, segmented, and fused all layers of the lesions for a layer by layer display, followed by merging them into the 3D volume of interest (VOI) (Fig. 1a,b). In case of a disagreement, the two doctors had a discussion with each other until reaching a consensus. Given the density of GIST and the characteristics of the enhancement technique, the venous phase was selected for tumor delineation in order to identify the tumor boundary and avoid error.

#### Extraction of radiomics features

The ROI file and the original of image of the segmented tumor were synchronously imported into the AK software in order to calculate the parameters of quantitative radiology features, including VOI. A total of 396 features of 5 categories, including the histogram feature, morphology feature, gray level co-occurrence matrix feature, run length matrix feature, and gray area size matrix feature were extracted for each patient.

#### Feature pre-processing

The extracted features were pre-processed before feature screening, as follows: (1) the median number of the feature was used when the omissive or abnormal values developed. (2) The dataset was randomly divided into the training group (n = 205) and the test group (n = 87) in the ratio of 7:3. The data of the training group were used for model construction, while those of the test group were used to validate the model accuracy and test its generalization power. (3) The features were standardized with the Z-score (minus the mean value divided by the standard deviation) in order to eliminate the interference between the feature dimensions.

#### Feature screening and modeling

For the training group, Spearman correlation analysis was first conducted in order to calculate the redundancy among feature parameters, and the threshold value of 0.9 was set to eliminate the strongly correlated feature parameters and only one of them was retained. Next, tenfold cross validation was performed via LASSO regression, and the features with non-zero coefficients were selected. To detect the multi-collinearity between variables in the combined model, the variance inflation factor (VIF) was used to perform the collinearity diagnosis with the VIFs > 5 indicating a severity collinearity.The parameters of the selected features were used to establish the model for GISK risk grading through logic regression analysis.

### Statistical analysis

The R (Version: 3.4.4) and SPSS (SPSS23.0) software were used for statistical analyses. The Kolmogorov–Smirnov test was performed to determine whether the data obeyed normal distribution. The t-test (normal distribution) and the Mann–Whitney U (skewed distribution) test were employed for comparison between the two groups of data. The data were represented as mean ± standard deviation (x ± s). The χ^2^ test was used to compare the data between the two groups of patients. The parameters such as accuracy, sensitivity, specificity, and area under curve (AUC) were used to evaluate the predication power of the model.

### Ethical approval

All procedures performed in studies involving human participants were in accordance with the ethical standards of the institutional (Huzhou Central Hospital, Affiliated Central Hospital Huzhou University) and/or national research committee and with the 1964 Helsinki declaration and its later amendments or comparable ethical standards.

### Informed consent

Informed consent was obtained from all individual participants included in the study.

## Results

### General demographics

A total of 292 patients (men: 140, women: 152, age: 29–90 years; average age: 61 ± 12 years) were categorized into the low-risk group (n = 127; very-low-risk cases: 32, low-risk cases: 95) and the high-risk group (n = 165; intermediate-risk cases: 46, high-risk cases; 119). A comparison between the general information of the two groups is listed under Table 1. No significant difference was noted with respect to the patient gender between the two groups. However, difference in the onset age exhibited a certain degree of significance. For instance, the onset age in the high-risk group was greater than that in the low-risk group. No significant difference in the degree of tumor enhancement was noted between the two groups, although the parameters of maximum tumor diameter and tumor morphology showed certain statistical significance.

**Table 1:** A comparison between the general information of the two groups.

Group	Cases	Gender		Age (x ± s)	Maximum tumor diameter (cm, ${\bar{\text{x}}}$ ± s)	Tumor morphology		Enhancement degree	
		Male	Female			Quasi-circular	Irregular	Significant	Insignificant
Low-risk	127	54	73	59 ± 10	2.6 ± 1.4	119	8	115	12
High-risk	165	74	91	63 ± 12	7.1 ± 4.1	133	32	158	7
Validation value		0.158^a^		− 3.133^b^	− 12.163^c^	10.409^a^		3.198^a^	
*P* value		0.722		0.008	0.000	0.001		0.061	

^a^ χ^2^ value, ^b^ t value, ^c^ Z value.

A clinical model for GIST risk grading was constructed with age, maximum tumor diameter and tumor morphology, which showed an AUC of 0.718 (95%CI: 0.659–0.776), with the sensitivity, and specificity of 66.1% and 61.9%.

### Radiomics model

**Figures 2:**
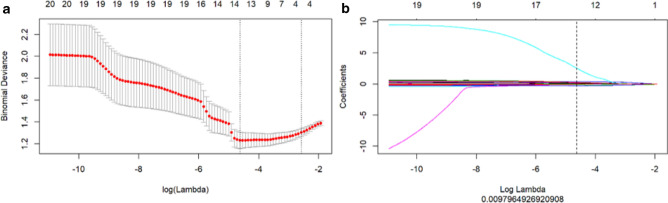
(**a**) Screening of the radiomics features was performed through LASSO regression. The cross-validation for LASSO regression, where the parameter λ was adjusted to find the best function set, is shown. The vertical dotted line on the left panel represents the log(λ) corresponding to the optimal λ. The selection criterion was the minimum deviation value, i.e. -4.3. (**b**) Screening of the radiomics features was performed through LASSO regression. The coefficients of texture parameters changed with λ. The vertical line corresponds to the 10 features selected with non-zero LASSO cross-validation coefficients.

A total of 396 quantitative imaging feature parameters were extracted using the AK software, and the inter-feature redundancy was first eliminated with the Spearman method to yield 20 features. These features were screened with the LASSO regression, and 10 features with higher predicative values were retained (Fig. 2a,b), which included 2 morphology features, 6 Gy-level co-occurrence matrix features, and 2 run-length matrix features. The feature parameters and their corresponding coefficients are listed under Table 2. All VIF values were less than 5. The radiomics score for each patient was calculated with the following formula:

**Table 2:** Texture parameters after the dimensionality reduction.

	Parameter		Coefficient	VIF values
Morphology features	Feature 1	Surface volume ratio	− 0.091	3.961
	Feature 2	Volume	2.535	2.384
GLCM feature	Feature 3	Inertia_angle135_offset1	− 0.298	1.452
	Feature 4	Sum Entropy	0.368	1.042
	Feature 5	HaralickCorrelation_AllDirection_offset1_SD	0.234	1.819
	Feature 6	GLCMEnergy_AllDirection_offset4_SD	− 0.076	1.431
	Feature 7	Correlation_AllDirection_offset7	0.104	4.027
	Feature 8	Correlation_angle135_offset7	0.076	3.098
RLM feature	Feature 9	LongRunHighGreyLevelEmphasis_angle0_offset1	0.051	1.929
	Feature 10	LongRunLowGreyLevelEmphasis_angle135_offset1	0.031	0.098

Rad score = 0.676 − 0.091 × feature 1 + 2.535 × feature 2 − 0.298 × feature 3 + 0.368 × feature 4 + 0.234 × feature 5 − 0.076 × feature 6 + 0.104 × feature 7 + 0.076 × feature 8 + 0.051 × feature 9 + 0.031 × feature 10.

**Figure 3:**
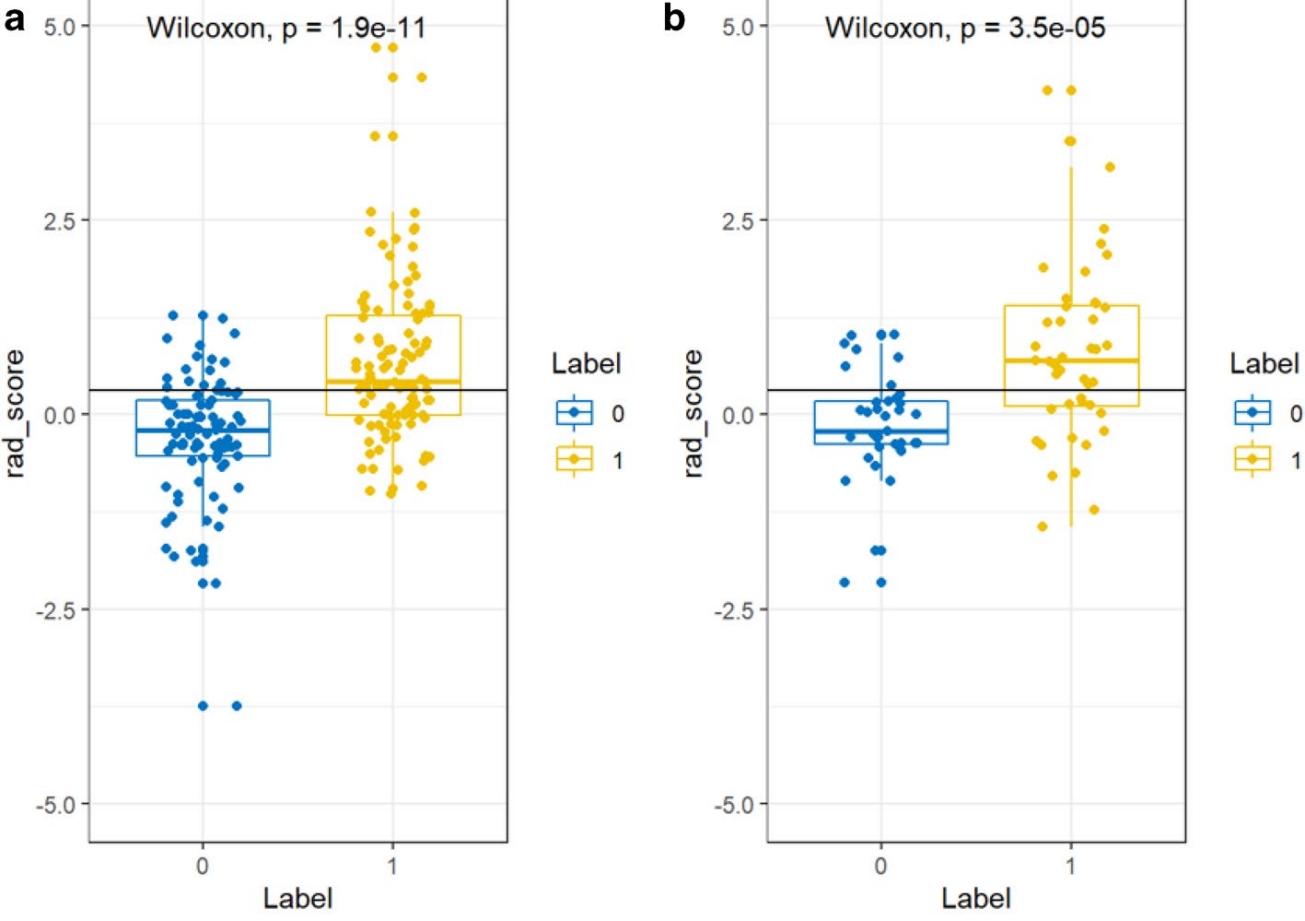
Radiomics score distribution of the 292 patients is shown for (**a**) the training group and (**b**) the validation group. The low-risk group is colored blue (0), and the high-risk group is colored yellow (1).

Statistical significance was recorded for the rad score in differentiating between the high- and low-risk GIST for both the training and test groups, and the rad score was found to be higher in the high-risk group than in the low-risk group (Fig. 3a,b).

**Figures 4:**
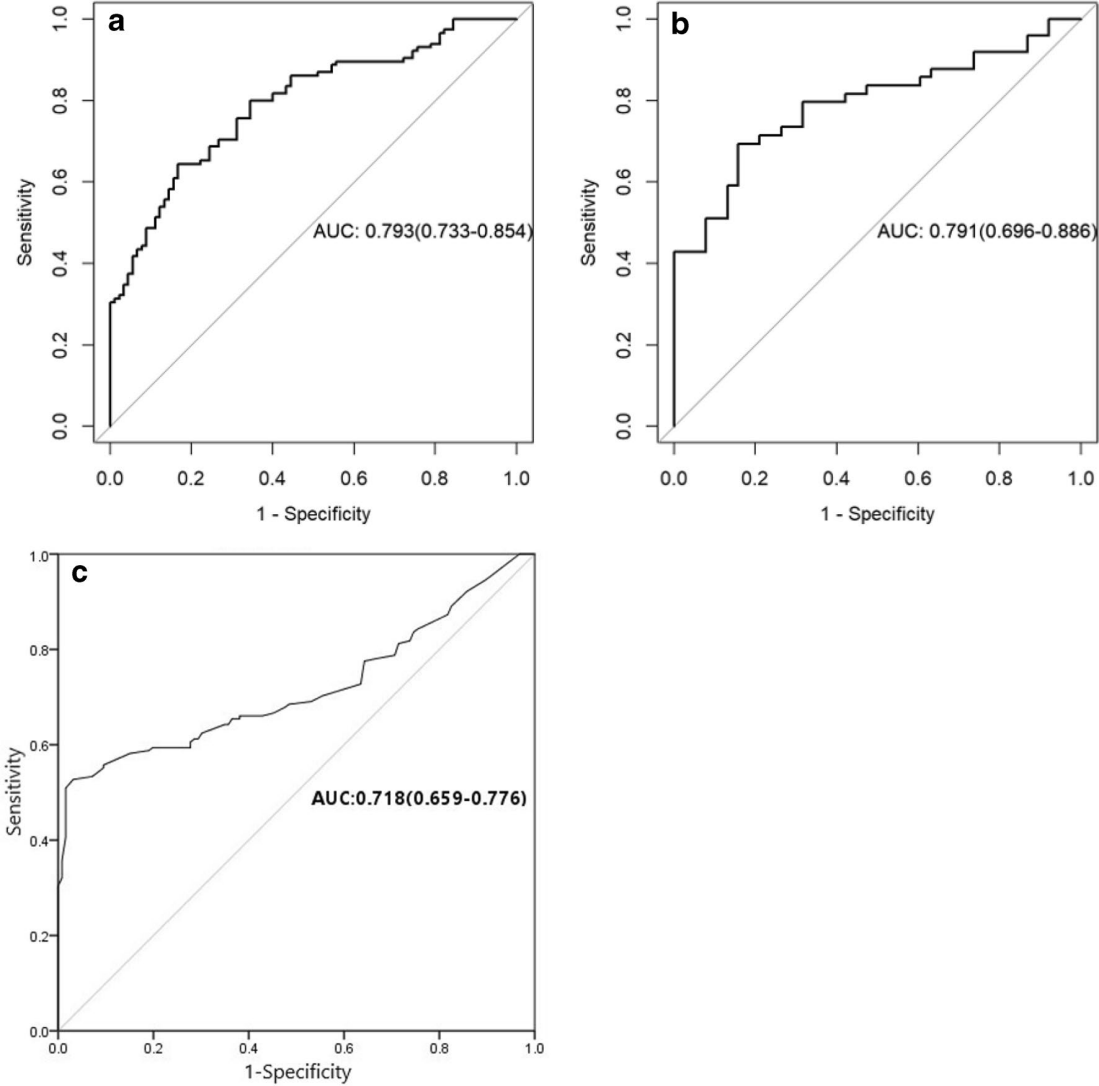
Evaluation of the radiomics model and clinical model for predicating GIST risk grading using the ROC curve. (**a**) It shows that the model’s AUC was 0.793 (95%CI: 0.733–0.854) for the training group (n = 205). (**b**) It shows that the model’s AUC was 0.791 (95%CI: 0.696–0.886) for the training group (n = 87). (**c**) It shows that the model’s AUC was 0.718 (95%CI: 0.659–0.776) for the clinical features (n = 292).

A predicative model for GIST risk grading was constructed from these feature parameters via the logic regression analysis. For the training group, the model showed an AUC of 0.793 (95%CI: 0.733–0.854), with the sensitivity, specificity, and accuracy of 83.3%, 64.3%, and 72.7%, respectively. For the validation group, the model’s AUC was 0.791 (95%CI: 0.696–0.886), with the sensitivity, specificity, and accuracy of 84.2%, 69.3%, and 75.9%, respectively. The cumulative results indicated that the proposed model possesses better predicative power than the clinical model (Table 3; Fig. 4a–c).

**Table 3:** Diagnostic efficacy of the radiomics model in the training and test groups.

	AUC (95%CI)	Accuracy (95%CI)	Sensitivity	Specificity
Train	0.793 (0.733–0.854)	0.727 (0.660–0.787)	0.833	0.643
Test	0.791 (0.696–0.886)	0.759 (0.655–0.844)	0.842	0.694

## Discussion

### The study features

With the recent development of artificial intelligence, radiomics methods have witnessed extensive application in disease diagnosis. Radiomics can extract feature data from images in a high-throughput manner that can be quantitatively analyzed for the transformation of the conventional medical images into quantitative data^8–12^. The results of this study yielded from the analysis of these data combined with the clinical and pathological information can be together used to guide clinical practices. As GIST risk grading is crucial for deciding the best clinical therapeutic option, and enhancement CT is advantageous owing to its convenience of use, intuitiveness, and non-invasiveness, the proposed approach serves as an important method of pre-surgical auxiliary examination^13–17^.

In the present study, ROI delineation is a premise for feature extraction. Segmentation of tumor ROI influences the feature extraction directly, where some features are extremely sensitive to the segmentation boundary. Past studies have demonstrated that the local tumor components cannot fully represent the tumor in general and that analyses based on the global domains of tumors can indicate tumor heterogeneity in a more accurate and reliable manner^8,12,16^. Therefore, in the current study, all layers containing the tumor parenchyma in the venous phase images of the enhanced CT were delineated layer by layer and then fused into a three-dimensional (3D) structure in order to ensure the generality and accuracy of the extracted feature parameters.

In addition to the conventional first-order histogram and morphology features, we employed the AK software to mine high-order texture parameters that are richer inside the tumors. The internal texture of GIST with different risk grading is mixed and locally irregular, but globally regular grey characteristics in the conventional CT images cannot be visually distinguished. These high-order texture parameters quantify the feature of gray level change within the tumor area and between the adjacent organs for the quantitative analysis of the tumor^17^, which provides additional valuable information for the auxiliary diagnosis or prognosis prediction.

### Analysis of feature parameters

In this study, after feature screening, LASSO retained two morphology features: surface-volume ratio and volume. The morphological features mainly described the 3D size and the shape of tumors^18,19^, among which the surface-volume ratio equaled to the surface of the VOI divided by the volume, while the tumor volume was calculated by multiplying the pixel numbers of the tumor area by the pixel size, which provided information about the lesion size. Similar to previous research^20^, our analysis showed that there was significant difference in age, maximum tumor diameter and tumor morphology between the two groups. The onset age in the high-risk group was greater than that in the low-risk group, so we speculated that higher grade tumors may present later in life. Among the final 10 features selected for model construction, the coefficient of volume was the largest, which indicated that the lesion size and the morphology of GIST were closely related to risk grading. The larger the volume, the smaller the SurfaceVolumeRatio, the higher the risk of GIST. These observation were further backed by the fact that only the parameters of maximum tumor diameter and tumor morphology exhibited statistical significance in the analysis of the general patient information.

The gray level co-occurrence matrix(GLCM) represents the joint probability of some pixel sets with a certain gray value^11^. The GLCM Correlation reflects the local gray level correlation in the image. Nine GLCM parameters (such as inertia, total entropy, full angle energy, correlation, and full-angle diagonal correlation) are included in the feature-screening results. These features describe the 2D spatial distribution of gray intensity^10,19^ that suggest, from another perspective, that the differences in the inner texture of tumors are important factors that influence tumor risk grading. The more uniform the GLCM matrix element value, the greater the correlation. The bigger the Inertia is, the higher the heterogeneity of pathological tissue; Entropy reflect inhomogeneity or complexity of the texture in the image, and its large value indicates a uniform texture pattern with regular changes. GLCMEnergy is the opposite, it reflects the uniformity of the gray distribution. LongRunHighGreyLevelEmphasis and LongRunLowGreyLevelEmphasis is the RLM features, which describe the roughness and smoothness of the image^8,12,17^. In this study, the Volume, Entropy, Inertia and LongRunHighGreyLevelEmphasis in high-risk GIST were greater than low-risk GIST, which indicated that the more heterogeneous in enhanced CT imaging, the greater the possibility of high-risk. The Correlation, GLCMEnergy in high-risk GIST were less than low-risk GIST, which indicated that the more uniform the pixel value and the more uniform the gray distribution. Among all selected parameters in this study, the numbers of gray level co-occurrence matrix and the run length matrix were the largest, which indicates that the texture distribution and spatial heterogeneity inside the tumors are closely related to tumor differentiation.

### Limitations

This study has several limitations. First, in this study, ROI was selected only from the solid portion of the tumor instead of from the calcified, hemorrhagically necrotic portion inside the tumor, thereby ignoring the significance of these special components in distinguishing tumors. Second, other important clinical indicators of GIST, such as the Ki67 expression, were not studied. Finally, the practicability of the prediction model warrants further verification by the big data and multi-institutional validation to be collected from prospective studies.

## Conclusions

The CT-enhanced radiomics model proposed in this paper demonstrated a good predicative power with significant potential value for the evaluation of the GIST risk status.
